# Role of the Interplay Between the Internal and External Conditions in Invasive Behavior of Tumors

**DOI:** 10.1038/s41598-018-24418-8

**Published:** 2018-04-13

**Authors:** Youness Azimzade, Abbas Ali Saberi, Muhammad Sahimi

**Affiliations:** 1 Department of Physics, University of Tehran, Tehran, 14395-547 Iran; 2 Institut für Theoretische Physik, Universitat zu Köln, Zülpicher Strasse 77, Köln, 50937 Germany; 3 Mork Family Department of Chemical Engineering Materials Science, University of Southern California, Los Angeles, California 90089-1211 USA

## Abstract

Tumor growth, which plays a central role in cancer evolution, depends on both the internal features of the cells, such as their ability for unlimited duplication, and the external conditions, e.g., supply of nutrients, as well as the dynamic interactions between the two. A stem cell theory of cancer has recently been developed that suggests the existence of a subpopulation of self-renewing tumor cells to be responsible for tumorigenesis, and is able to initiate metastatic spreading. The question of abundance of the cancer stem cells (CSCs) and its relation to tumor malignancy has, however, remained an unsolved problem and has been a subject of recent debates. In this paper we propose a novel model beyond the standard stochastic models of tumor development, in order to explore the effect of the density of the CSCs and oxygen on the tumor’s invasive behavior. The model identifies natural selection as the underlying process for complex morphology of tumors, which has been observed experimentally, and indicates that their invasive behavior depends on both the number of the CSCs and the oxygen density in the microenvironment. The interplay between the external and internal conditions may pave the way for a new cancer therapy.

## Introduction

Cancer usually begins with out-of-order duplication of a single cell that has stem cell-like behavior, referred to as the cancer stem cell (CSC)^1^. Based on the CSC hypothesis, a CSC can duplicate without limit and differentiate^2^. The classical CSC hypothesis proposes that, among all cancerous cells, only “a few” act as stem cells, but studies have reported^3^ that a relatively high proportion of the cells are tumorigenic, contradicting the general belief. The CSCs have been proposed as the driving force for tumorigenesis and the seeds for metastases^4^. Their decisive role in maintaining capacity for malignant proliferation, invasion, metastasis, and tumor recurrence has been reported frequently^5^. For example, CSCs of breast tumor are involved in spontaneous metastases in mouse models^6^. Moreover, CSCs promote the metastatic and invasive ability of melanoma^7^ and their prsence is correlated with invasive behavior at colorectal adenocarcinoma^8^. The effect of the number of CSCs on tumor morphology has been the subject of several experimental studies and simulation. Based on simulations^9,10^, the frequency of the CSCs smoothens the morphology of tumor, and based on an experimental study^11^, the number of CSCs is higher in tumors with medium invasiveness (the so-called Gleason grade) than tumors with lower (Gleason grade) and higher (Gleason grade) invasiveness. The relation between tumor malignancy and the frequency of the CSCs needs, however, more clarification^4^.

Cancerous cells use oxygen to produce metabolites for duplication and growth^12^. Experimental *in-vivo*^13^ and *in-vitro*^14^ studies, as well as computer simulations^15,16^, have reported that the density of oxygen regulates tumor morphology and its shortage drives morphological irregularities. Due to the apparent strong correlations between the tumors’ shape and their malignancy, fractal characterization of tumors has been used as a diagnostic assay for various types of tumors^17–19^. However, there is still no explanation as to why cellular structures at the scale of tumors display self-similar characteristics^20^. of well-known physical phenomena, including diffusion and reaction-diffusion, as well as percolation, surface growth, and models of phase transition^21^.

In this paper we propose a novel model to study the effect of the number of the CSCs and the oxygen’s density on the invasive behavior of a general type of cancer. As we show below, the development of irregular shapes and the respective tumor’s invasive behavior are correlated with the two factors. Unlike the previous studies, we present a quantitative measure by which one can understand better the effect of completion on the malignancy of tumors. We take the shape irregularity as the factor for identifying the invasive behavior of tumor and compare our results with experimental reports. The model that we present contains the essential features of the cells, such as symmetric/asymmetric division, metabolic state, cellular quiescence and movements, apoptosis, and existence of oxygen and its consumption. Our results explain, for the first time to our knowledge, the aforementioned experimentally-observed fractal behavior and contradict the predictions of recent models for the relation between the number of the CSCs and the growth rate and invasion. In addition, we believe that the results may cast doubt on the recent therapeutic approach based on oxygen deprivation.

## Results

**Figure 1:**
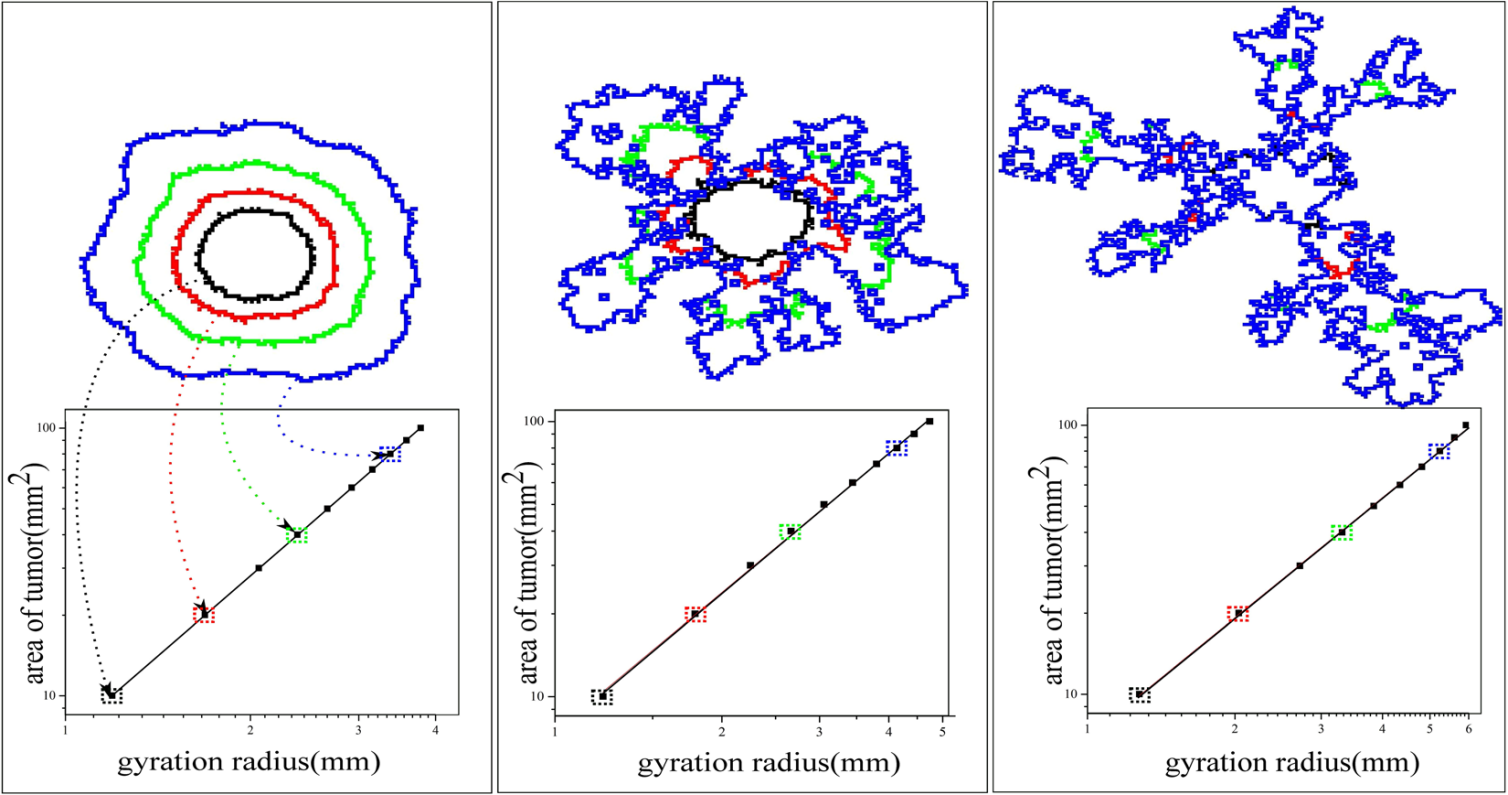
Fractal structure of the tumors. Tumors are irregular, but exhibit self-similarity. The linearity of the plot indicates fractal behavior, with the slope being *D*_*f*_ ≈ 1.99 ± 0.01 for *p*_*s*_ = 0.1 (left), 1.76 ± 0.02 for *p*_*s*_ = 0.5 (middle), and 1.47 ± 0.02 for *p*_*s*_ = 1 (right), with (normalized) oxygen density, *n* = 1. Each contour line represents the borderline of the tumor with the corresponding gyration radius indicated by the dotted arrows. It should be noted that the figure on the left covers 5000 units (50 mm^2^) in 5000 time steps (30 days), while the ones in the middle and right cover the same area in 14000 (55 days) and 12000 (83 days) steps, respectively. The simulations were carried out in a 200 × 200 lattice

As the system evolves, the cells consume oxygen, enhance their metabolic state, and proliferate after reaching the energy level of *u*_*p*_, in order to create a clone - the tumor - see Fig. 1. The perimeter of the clone is the main object that we study in this paper.

As Fig. 1 demonstrates, the cells take on irregular shapes during their growth whose complexity depends on the number of the CSCs (or probability *p*_*s*_). One interesting approach is to study the structure of the perimeters in the context of interface instability^22–24^. The analogy with the instability of interfaces has been established for the case of melanoma^25^, and the instabilities were attributed to nutrient density. But, here, we quantify tumor behavior through classifying irregular morphology of the tumors. To quantify the irregularity of the tumor’s morphology and its evolution, we use fractal analysis. To this end, we measure the average distance *r* from the center of the mass, as well as the area of the tumor during its growth. Figure 1 indicates that log(area) versus log *r* is a linear plot so that, ${\rm{area}}\sim \,{r}^{{D}_{f}}$. Thus, the slope of the line in the logarithmic plot is the fractal dimension *D*_*f*_, implying self-similarity of the tumors of various sizes. The self-similarity of the tumors’ growth is the result of heterogeneous duplication on their perimeter, which itself is due to the oxygen gradient. Cells in the region with higher curvatures have better supply of oxygen, helping them increase their metabolic state, and proliferate faster. The proliferation also creates new perimeter curvature with the same behavior. As the number of oxygen consumers, which is proportional to *p*_*s*_, increases the competition between the cells for the limited oxygen supply intensifies and oxygen availability becomes more heterogeneous. Thus, the tumors take on more irregular shapes or lower fractal dimension *D*_*f*_, contradicting previous studies^9,10^ that proposed an adverse relation between the number of the CSCs and the invasive behavior.

We note that fractal scaling has been reported previously in the experimental studies^17,18^. Moreover, irregular shapes have been interpreted as an indication of invasive behavior of various tumors^17–19^. Tumors with more irregular shapes are more invasive, and in our model the more irregular tumors have smaller *D*_*f*_. There are several reports that confirm the correlation between *D*_*f*_ and tumor malignancy (a malignant tumor possesses a lower fractal dimension than that of a benign mass)^26–29^.

**Figure 2:**
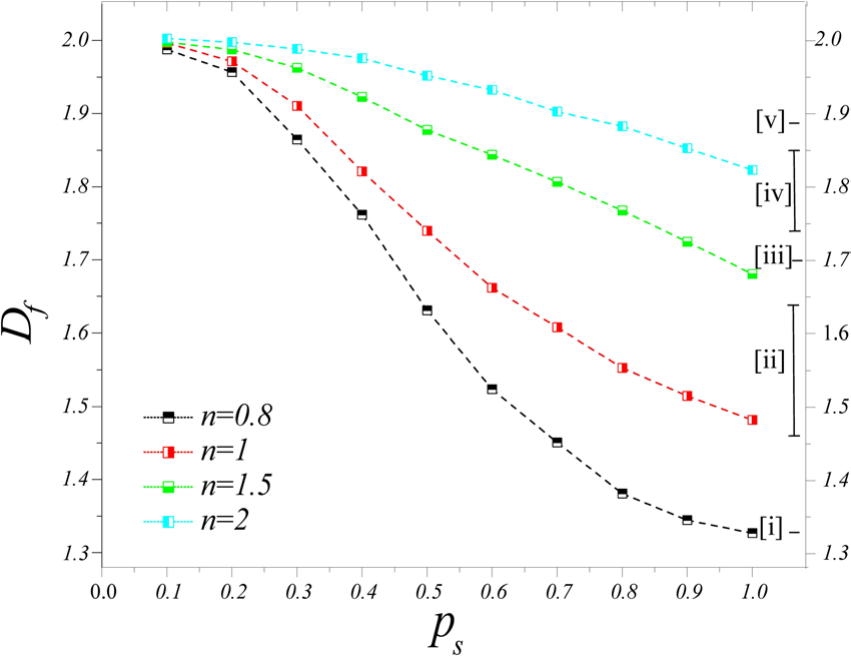
Interrelationship between malignancy, immortality and oxygen density. Fractal dimension *D*_*f*_ as an indication of malignancy for various tumors. Our model reproduces some of the previously reported fractal dimensions: [i] ${D}_{f}\sim 1.338\pm 0.248$^30^; [ii] 1.46 ¡ *D*_*f*_ ¡ 1.64^29^; [iii] ${D}_{f}\sim 1.696\pm 0.009$; [iv] 1.74 ¡ *D*_*f*_ ¡ 1.85^31^, and [v] ${D}_{f}\sim 1.887\pm 0.008$^32^.

A study of the variations of *D*_*f*_ with *p*_*s*_ and the density *n* of the oxygen is useful to characterization of the tumor behavior. The computed *D*_*f*_ for various values of *p*_*s*_ and oxygen densities is shown in Fig. 2.

Figure 2 presents explicitly the value of *D*_*f*_ and the corresponding malignancy of tumor as a result of both the internal feature and the external conditions. For a fixed density *n* of oxygen, the invasive behavior of tumor always increases with *p*_*s*_, implying that, regardless of the environmental conditions, higher numbers of CSCs always lead to a more invasive behavior; see Fig. 2 in the Supplementary Information (SI). This is in contradiction with the existing reports on the adverse effect of *p*_*s*_ on the tumor’s invasive behavior^9,10^. On the other hand, the effect of the environmental stress on invasion is regulated by internal feature of the cells, *p*_*s*_. For *p*_*s*_ = 1, the oxygen deprivation significantly increases the malignant behavior of tumors, while for *p*_*s*_ = 0, the density of oxygen has negligible effect on tumor’s invasive behavior.

## Relation to Superficial Spreading Melanoma

As presented here, our model explains a two-dimensional (2D) tumor growth. Early stages of Superficial Spreading melanoma has a 2D structure that might be a promising case to apply our findings to. Experiments indicate that there is no blood flow to the Superficial Spreading melanoma (SSM) with thickness less than 0.9 mm^33^. In addition, melanoma is, at least in its early stages, an approximately 2D phenomenon, so that a 2D model may properly produce its structure. The malignant cells in SSM stay within the original tissue - the epidermis - in an *in-situ* phase for a long time, which could be up to decades. Initially, the SSM grows horizontally on the skin surface, known as *radial growth*, with lesion indicated by a slowly-enlarging flat area of discolored skin. Then, part of the SSM becomes invasive, crossing the base membrane and entering the dermis, giving rise to a rapidly-growing nodular melanoma within the SSM that begins to proliferate more deeply within skin.

## Discussion

The proposed model sheds new light on and provides new insight into the invasive behavior of tumors by deciphering the effect of both intrinsic and extrinsic features of cells. It also demonstrates that elimination of the oxygen in the previous models gives rise to such a relation. The fractal behavior that we identify and attribute to the growth limited to the perimeter is similar to surface growth^17,34^. Nevertheless, close inspection of the proliferation activity in the perimeter in the proposed model reveals larger parts of the cells as proliferative cells; see Fig. 1 of the SI. As the model demonstrates, a single biological parameter, namely *p*_*s*_, changes the cell’s features and results collectively in various self-similar states with distinct fractal dimensions. Previous models, which considered the CSCs^9,10^, obtained an inverse relation between the number of the CSCs and invasion, but our model indicates increased malignancy to be proportional to larger numbers of the CSCs. Compared to experimental data^11^ our model confirms increasing of morphological irregularities (Gleason grade), but complete consistency require more biological details in the model.

Tumors with low number of the CSCs that were proposed by the previous studies^9,35^ did not respond to oxygen deprivation, as was expected^13,14^. Hence, tumors that respond to oxygen deprivation must have larger number of the CSCs. In addition, models that do not consider the CSC evolution and endow the cells with unlimited proliferation capacity^14,15^ produce tumors corresponding to *p*_*s*_ = 1. Such models consider the effect of oxygen and, as our model confirms, oxygen deprivation leads to higher irregularities. As *p*_*s*_ decreases, the effect of oxygen vanishes. Thus, a lower number of the CSCs, which was proposed previously^9,35^, does not conform to the experimentally well-established oxygen effects. Our model, in addition to reproducing such result, provides quantitative and comparable results to classify the irregularities that can be used to analyze experimental data that have been reported for the fractal dimensions.

The conceptual results are applicable to the growth of other solid tumors that display the aforementioned behavior in response to oxygen tension and frequency of CSCs. For example, in the case of the SSM in which the number of CSCs is not small^3,36^, oxygen deprivation probably increases tumor malignancy. Contrary to the previous studies, the present model predicts invasion as the result of *both* the tumor *and* the microenvironment, demonstrating the effect of nutrient deprivation on the invasion. This implies that recent studies on such therapeutic approach^37,38^ must consider carefully the side effects that, based on our model for tumors with larger numbers of the CSCs, can increase tumor malignancy.

## The Model

**Figure 3:**
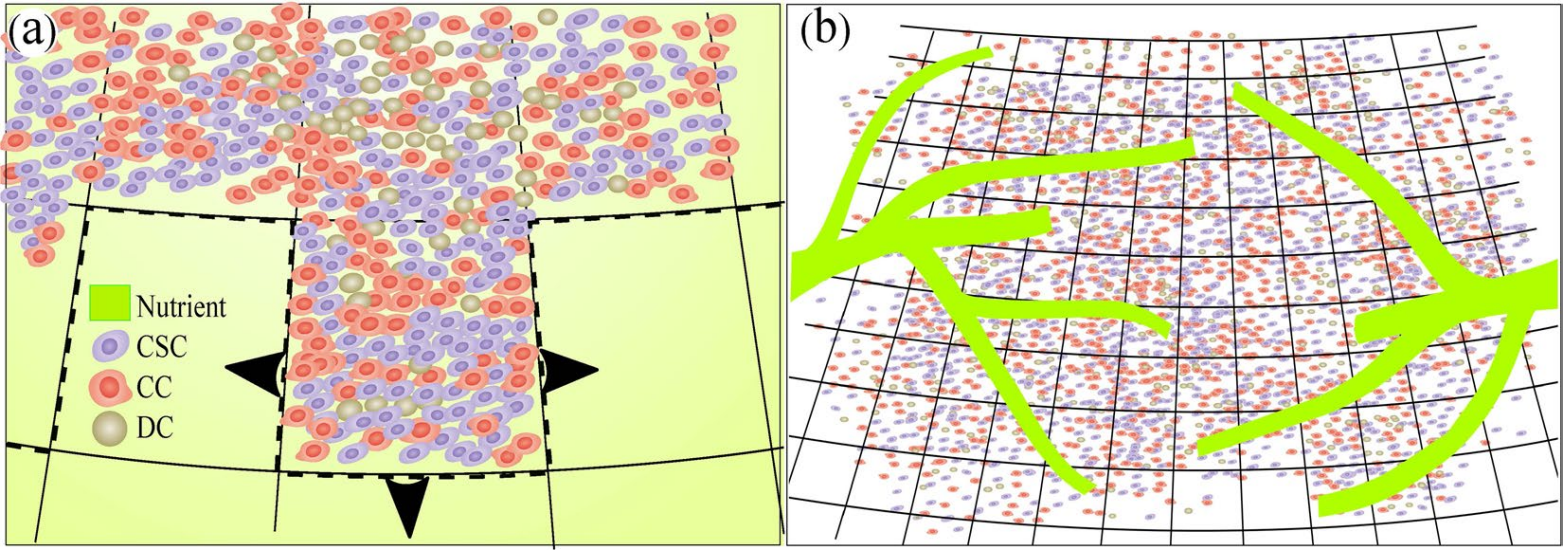
Schematic of the model. (**a**) Various types of cells that are either proliferating or dying. Nutrient density in the milieu is constant and after diffusing from the surrounding is consumed by the cells. (**b**) An alternative mechanism for oxygen supply by the capillaries coming from the third dimension to feed the tumor at random sites. The results do not depend on the choice of the initial/boundary conditions for the nutrient; see the SI.

Similar to many other natural systems, biological media fluctuate due to the intrinsic randomness of the individual events^39^. Cells are involved in regulatory pathways that depend highly nonlinearly on the chemical species that are present in low copy numbers per cell^40^, as a result of which other factors, such as the forces between cells, fluctuate significantly^41^. Thus, statistical approaches are suitable for simulating cells’ behavior. We consider the 2D lattice shown in Fig. 3 in which each bond is 100 micrometer long, while each site has the capacity for 100 cancer cells that typically have 10 *μ*m diameter^42^. The nutrient density is constant on the perimeter of a circle with a radius of 1 cm. It diffuses into the internal zones and is consumed by the living cells. In the SI we present the results for various other initial/boundary conditions for the oxygen supply, including smaller and larger radii of the circle, regular and random distribution of the oxygen source, as well as its uniform distribution in the medium, and show that the predictions of the model do not depend on the choice of the oxygen supply mechanism. Though we consider 2D structures, the results for a 3D structure for oxygen supply system (vessels and capillaries) would remain qualitatively the same, while the model can be extended to 3D.

Keeping the oxygen density uniform in the milieu −0.15 mol/ml^16^ - a CSC is inserted at the center of the medium that consumes the oxygen and enhances its metabolic state. Although metabolic pathways are not fully understood, metabolic activity is a crucial factor in a cell’s decision to either proliferate or die^43^. In the former case a cell must increase its biomass and replicate its genome prior to division, in order to create two daughter cells. Thus, the cell must generate enough energy and acquire or synthesize biomolecules at a sufficient rate to meet the proliferation demand^44^. Given such biological facts, we choose metabolic state as the decisive factor for a cell’s decision to proliferate, and define an internal energy *u*_cell_ for each cell as an indicator of its metabolic state. Physically, the cells acquire energy from the environment to accumulate internal energy^45^ - the energy of the absorbed molecules - which evolves according to the energy conservation law:

1 $$\frac{\partial {u}_{{\rm{cell}}}}{\partial t}=\chi n(x,y,t)-\gamma {u}_{{\rm{cell}}},$$

where *n*(*x*, *y*, *t*) is the oxygen density at position (*x*, *y*) and time *t*, with *χ* and *γ* being positive constants related to energy accumulation and consumption rate (for details of all the constants and their values see Table 1 in the SI). If a cell’s energy reaches a threshold *u*_*p*_, it will begin duplication. We set *u*_*p*_, *χ* and *γ* such that every cell in the appropriate situation will be in the duplication state after 15 hours^46^, which is about the time that tumor cells need to reach the so-called cell checkpoints *eG*_1_ (early *G*_1_), *G*_1_ and *eS* in the cell cycle for division. *G*_1_ is the primary point at which a cell must decide whether to divide. After it passes *G*_1_ and enters the *S* phase, the cell is committed to division^46^ (other checkpoints, such as *G*_2_ at which the cell is mostly concerned with the condition of its DNA, still remain to be completed in the next step). As we show below, Eq. (1) together with the limits imposed, reproduces cell plasticity and various proliferation activities under a variety of external conditions^47^ that were reported recently^46^. Time is measured in units of 10 minutes.

**Figure 4:**
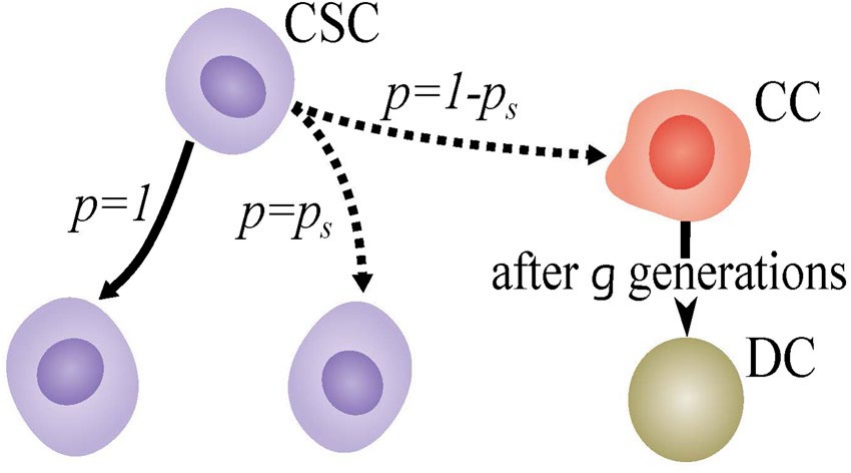
Division of the cells. During division each CSC creates another CSC. The other daughter cell would either be a CSC with probability *p*_*s*_ or a CC with probability 1 − *p*_*s*_. Each CC creates two CCs during duplication, if it is capable of division. The CSCs can continue the division process for a long time, whereas each CC loses its ability for duplicating after *g* divisions, and dies. Clearly, the first CC daughters could duplicate *g* − 1 times, where we set *g* = 5^10^.

The evolution of the internal energy *u*_cell_ of the cells depends on the local density of oxygen through a set of coupled differential equations, and if enough oxygen exists at the position of the first CSC, *u*_cell_ increases to *u*_*p*_ and the first CSC duplicates into two daughter cells. This relation between oxygen density, cell metabolic state and its duplication dynamics ensures the apparent role of the oxygen density in the tumor evolution. One may consider various scenarios for quantitative studies of the CSC proliferation^48–51^, but the probability of distinct kinds of divisions has yet to be assessed experimentally. Besides, some other studies^52^ have proposed the cells’ self-renewal ability as the prerequisite for tumor maintenance. Thus, we choose the simplest biologically-correct model that has the ability to generate the entire possible range of the CSC population percentage, from zero up to the values produced by the various mathematical^48–51^ and biological models^52^. In this model, during duplication of each CSC one daughter cell is assumed to be CSC, while the second one is either a CSC with probability *p*_*s*_ - the probability of symmetric duplication of the CSCs - or a cancerous cell (CC) with probability (1 − *p*_*s*_); see Fig. 4. Each CC duplicates into two CCs if it is allowed to^10^. Such a probabilistic approach is motivated by the fact stated earlier, that according to the classical CSC hypothesis, among all cancerous cells, only “a few” act as stem cells, whereas some studies^3,53^ have reported that the population of CSCs can be relatively high, which is why we take the population of the CSCs (with probability *p*_*s*_) as a parameter of our model. For *p*_*s*_ = 1 the model reduces to the stochastic model of tumor development^54^. Every CSC continues such a division for an unlimited frequency, but the CC can have only limited generations of duplication^55^, which we set it to be *g* = 5^1,10^ after which it will die and produce dead cells (DCs); see Fig. 4. As the cells undergo apoptosis, they are recognized and removed from the body by phagocytes. Thus, we assume that the dead cells remain inactive in the medium, but even if we eliminate them after death, the main results remain the same; see the Fig. S15 in the SI.

We define the density of cells of type *i* at location (*x*, *y*) at time *t* by,

2 $${C}_{i}(x,y,t)=\frac{{\rm{number}}\,{\rm{of}}\,{\rm{cells}}\,{\rm{at}}\,(x,y,t)}{{\rm{capacity}}\,{\rm{of}}\,{\rm{each}}\,{\rm{site}}}\,,$$

with *i* ≡ CSCs, CCs, and DCs. Equation (2) is also valid for the total density of cells, *C*_*t*_ = *C*_CSC_ + *C*_CC_ + *C*_DC_. Recall also that the capacity of each site is 100 cells^42^. The density of the CCs is denoted by *C*_CC_(*x*, *y*, *t*; *j*) in which *j* indicates their generation that varies from 1 to *g* (after *g* generations they produce the DCs). Healthy tissues contain healthy cells in which the distribution of the nutrients is in a steady state. We eliminate the healthy cells for all the tumors, as our results are based on comparison with and differences of tumors’ behavior that are the most important part of our study.

Local density gradients drive the stochastic motion of the cells^56^. Thus, one has,

3 $$\frac{\partial C(x,y,t)}{\partial t}=D{\nabla }^{2}C(x,y,t),$$

where *D* is the diffusion coefficient. Equation (3) is applicable to the various kinds of cells, for which^16,57^ *D* ≈ 10^−10^ cm^2^/s. Population growth of biological groups depends on the species ability for proliferation and the environmental limitations. One important environmental limit is contact inhibition of cell division^58^, i.e., if after the energy rises to *u*_*p*_, the cells will duplicate if there is space; otherwise, they will stay quiescent until they find space for duplication^59^. Thus, proliferation at each site depends on the number of cells that can duplicate, and the effect of competition for space between all types of cells. The evolution of the CSCs that qualifies for the duplication metabolic threshold *u*_*p*_, is expressed by a diffusion-reaction equation,

4 $$\begin{array}{rcl}\frac{\partial {C}_{\mathrm{CSC}}(x,y,t)}{\partial t} & = & D{\nabla }^{2}{C}_{\mathrm{CSC}}(x,y,t)\\  &  & +\,{R}_{m}{p}_{s}{C}_{\mathrm{CSC}}(x,y,t\mathrm{)[1}-{C}_{t}(x,y,t)],\end{array}$$

where *R*_*m*_ is the rate of passing the *S*, *G*_2_ and *M* phases in the cell cycle, which is fixed as a cell that has enough internal energy (has passed the aforementioned *eG*_1_, *G*_1_ and *eS* phases) will duplicate in 5 hours^46^, if there were no other cells. The last term on the right side of Eq. (5) that includes the term [1 − *C*_*t*_(*x*, *y*, *t*)] captures the effect of contact inhibition of proliferation in which *C*_*t*_(*x*, *y*, *t*) is the total density of all cells at (*x*, *y*, *t*). The entire cell cycle takes 20 h. The evolution of the *j*th generation of the CCs is governed by

5 $$\begin{array}{rcl}\frac{\partial {C}_{{\rm{CC}}}(x,y,t;j)}{\partial t} & = & D{\nabla }^{2}{C}_{{\rm{CC}}}(x,y,t;j)\\  &  & +\,{\delta }_{1j}{R}_{m}\mathrm{[1}-{p}_{s}\mathrm{][1}-{C}_{t}(x,y,t)]\\  &  & +\,\mathrm{(1}-{\delta }_{1j}){R}_{m}{C}_{{\rm{CC}}}(x,y,t;j-\mathrm{1)[1}-{C}_{t}(x,y,t)]\\  &  & -\,\mathrm{(1}-{\delta }_{jg}){R}_{m}{C}_{{\rm{CC}}}(x,y,t;j\mathrm{)[1}-{C}_{t}(x,y,t)]\\  &  & -\,{\delta }_{jg}{R}_{a}{C}_{{\rm{CC}}}(x,y,t;j),\end{array}$$

where *δ*_*ij*_ denotes the Kronecker delta, i.e., *δ*_*ij*_ = 1 for *i* = *j* and 0 otherwise, with 1 ≤ *i*, *j* ≤ *g*. The first term on the right side of Eq. (5) represents diffusion of the cells due to the local concentration gradient;^16,56^ the second is the creation of the first generation of the CCS due to asymmetric duplication of the CSCs^10^, while the third term represents the creation of the *j*th generation (for *j* ≠ 1) of the CCs from duplication of the prior generation. The concentration of the CCs decreases due to duplication and creation of the next generation, which the 4th terms accounts for, while the last term takes into account the death of the final (*g*th) generation of the CCs. *R*_*a*_ is the rate of apoptosis - the process of programmed cell death - and is fixed as the *g*th generation has a halflife equal to 1 day. Finally, the evolution of the oxygen density in the presence of the cells is governed by

6 $$\begin{array}{rcl}\frac{\partial n(x,y,t)}{\partial t} & = & \beta {\nabla }^{2}n(x,y,t)\\  &  & -\,\alpha [{C}_{\mathrm{CSC}}(x,y,t)+\sum _{j=1}^{g}{C}_{{\rm{CC}}}(x,y,t;j)],\end{array}$$

with *α* being proportional to oxygen consumption rate by the cells, which is the same for both the CCs and cancerous stem cells. We varied the rates of oxygen consumption for every kind of cells, but the essential results remained the same; see the SI. *α* was fixed by setting the reported value for oxygen consumption^16,60^ to be 6.65 × 10^−17^ mol cell^−1^ s^−1^. *β* is the diffusion coefficient of oxygen in the medium, which we fixed it based on the calculations at room temperature, 10^−5^ cm^2^/s. We present in the SI the results for other values of *β*. For distances more than 1 cm from the medium’s center the oxygen density is constant (see the SI for the results for larger and smaller distances, as well as other ways of supplying the oxygen), and is equal to 0.15 mol/ml^16^. For simplicity, in all the calculations we normalize *n* to 1. From outside of the aforementioned circle, oxygen penetrates into the central area. Given the assumptions, the cells are active elastic species, consuming oxygen and proliferating.

As we show in the SI, other boundary conditions do not change the essential results. In addition, (i) we also varied both the proliferation activity and oxygen consumption rate for various kinds of cells, but the results remained qualitatively the same. (ii) The CSCs and CCs are assumed to have equal oxygen consumption rates, but when we changed them for every kind of cell, the results were qualitatively the same. (iii) The CSCs and CCs are assumed to have the same internal energy threshold *u*_*p*_ for duplication, and equal rates of crossing the *S*, *G*_2_ and *M* phases in the cell cycle. But changing the proliferation activity of the cells did not change our main results. Let us also emphasize that our model is not the same as the classical models of diffusion-limited aggregation^61^, as such model did not deal with the effect of reaction and consumption.

## Electronic supplementary material

Supplementary Information
