# Supplementary material for: Role of the Interplay Between the Internal and External Conditions in Invasive Behavior of Tumors
Source: Sci Rep. 2018 Apr 13;8:5968. doi: 10.1038/s41598-018-24418-8 (PMC5899171; doi:10.1038/s41598-018-24418-8)
Supplement: Supplementary file 1 — Supplementary Information [file 41598_2018_24418_MOESM1_ESM.pdf]

Supplementary Information for "Role of the Interplay Between the Internal and External Conditions in Invasive Behavior of Tumors "

Youness Azimzade,[1] Abbas Ali Saberi,[1, 2, *] and Muhammad Sahimi[3]

[1]*Department of Physics, University of Tehran, Tehran 14395-547, Iran*
[2]*Institut für Theoretische Physik, Universitat zu Köln, Zülpicher Strasse 77, 50937 Köln, Germany*
[3]*Mork Family Department of Chemical Engineering Materials Science, University of Southern California, Los Angeles, California 90089-1211, USA*

We first provide the details of the model described in the paper, and the procedure for the numerical simulations. The processes that we include in the model are, (i) diffusion of the nutrient from the environment toward the cells and its consumption by them; (ii) the cellular metabolic state and its effect on the cells behavior; (iii) proliferation of the cancerous cells according to the cancer stem cell (CSC) hypothesis, and (4) migration of the cells due to the concentration gradient in their population.

The model includes two main steps: (i) random selection of the sites on the lattice and their updating over time (by a Monte Carlo method), and (ii) numerical simulation of the partial differential equations governing the evolution of the *nutrient* [Eq. (4) of the text], the *internal energy* [in the case in which a cell is present at the site, Eq. (1)], and the cell concentration at the selected sites [Eqs. (2) and (3) in which the proliferation rate is conditioned upon fullfilment of the threshold condition u_p considered in the internal energy].

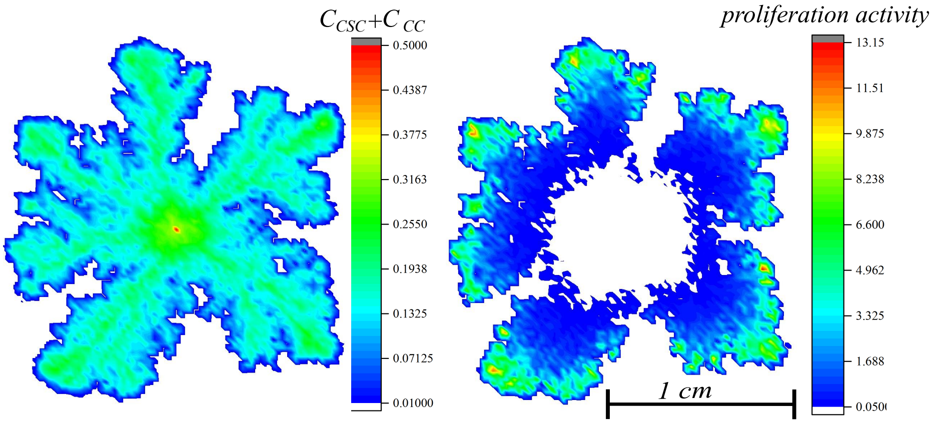

FIG. 1: Proliferation activity of tumor and concentration of the living cells. Cells proliferate if they can meet metabolic demands of proliferation and have enough space. It appears that such requirements are available for some internal cells, as well as those on the border. Active cells thickness reaches 3 mm in some area. The unit of proliferation activity at each site is the number of devisions in the previous 96 hours at that site, which can be a noninteger number because we consider proliferation as a continuous process.

The nutrient concentration is held fixed in the medium, and a CSC is placed at the central plaquette in the lattice. A lattice site is then randomly chosen and the governing equation, Eq. (4), for the diffusion and consumption of the nutrient for the living cells (if any) are first numerically solved. If there exist living cells at the chosen site (or plaquette), Eq. (1) for the internal energy is also solved numerically. If the internal energy of the cell exceeds the threshold u_p, then the cell will proliferate according to the rules summarized in Fig. 3 of the text. In the case of duplication of a CSC, Eq. (2) is numerically solved; otherwise, the CSC concentration is only governed by the first term of Eq. (2). After the first round of the proliferation develops, the first generation of the cancerous cells is produced [the second term in Eq. (3)]. Each "time step" is defined by the completion of 201×201 (the total number of sites of the lattice) trials, i.e. every site on average has the chance to be updated at least once. By increasing the internal energy of the cancerous cells, the next generations may emerge, leading ultimately to the production of the dead cells. Repeating the procedure over time, a tumor forms whose morphological properties are the main subject of the paper.

*Electronic address: ab.saberi@ut.ac.ir

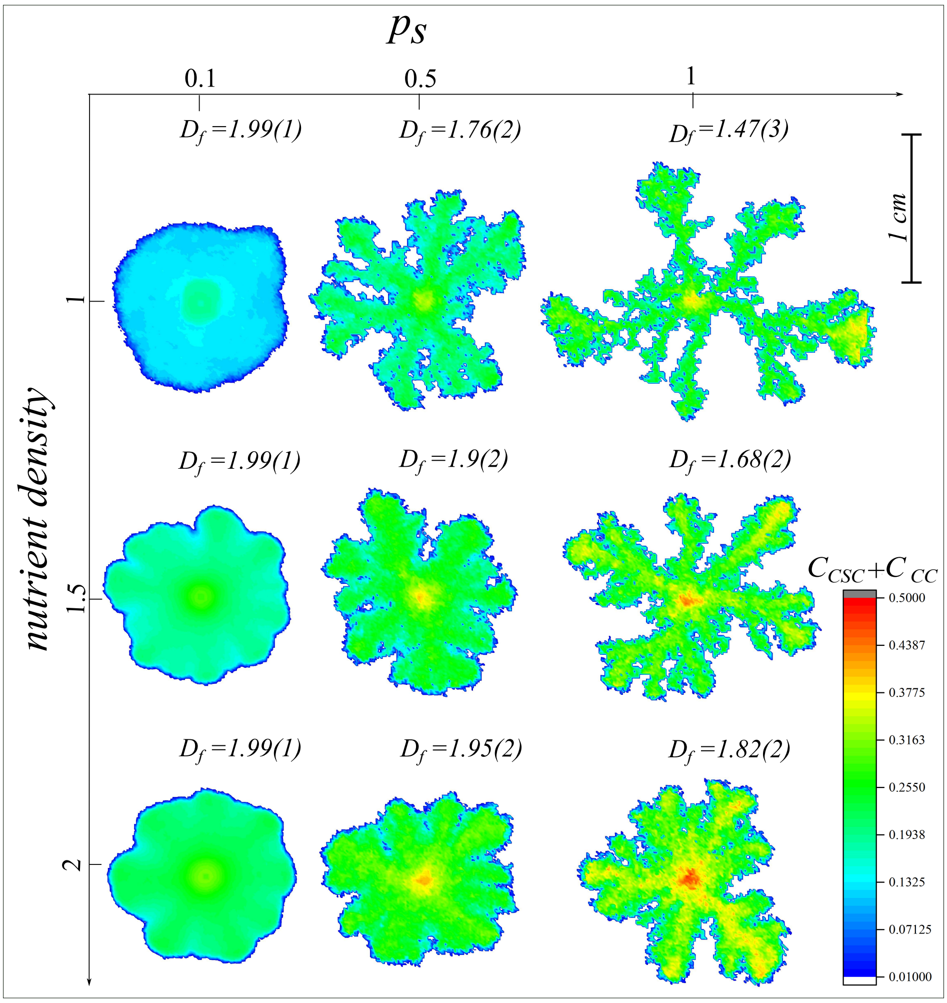

FIG. 2: Shapes irregularities and the corresponding fractal dimension D_f, which is averaged over 100 tumors with the same probability p_s and in the same environment.

The main parameters of the model are, (i) p_s, the cancerous stem cell proliferation rate, and (ii) n, the nutrient density in the medium. The competition between the two parameters, representing the internal and external factors, can lead to completely different structures for the tumors, an issue that was addressed in the past. We believe that this might pave the way to devise an experiment to test the CSC hypothesis, as well as help controlling the tumor growth by controlling the external parameters.

Note that the main idea of the paper is based on many experimental observations in which a qualitative relation between the complexity of the morphology of tumors and their malignancy was reported. In this paper we have quantified the relation in terms of two possible main parameters that may play major roles in the tumor growth.

As emphasized in the paper, proliferation is not confined to a tumor's border, as previously was suggested [1, 2]. About 200 layers of cells on the border contribute to proliferative activity that can barely be considered as "surface" growth.

The morphology of the tumors under the effect of various number of the CSCs and nutrient availability: In contrast to the previous studies [3–7], internal features of the tumors (in this case the number of the CSCs) can increase tumor malignancy. In addition to the fractal analysis, Figure 9 depicts the malignancy of tumors as a result of *both* the internal features *and* the external environment of the tumor.

Malignancy may be the result of distinct conditions. We chose the tumors with fractal dimension $D_f \approx 1.82$ as malignant. This type of tumors can arise by various values of p_s and n; see Fig. 10.

Circularity is another method for classifying irregular shapes based on their space-filling feature. It is a measure of

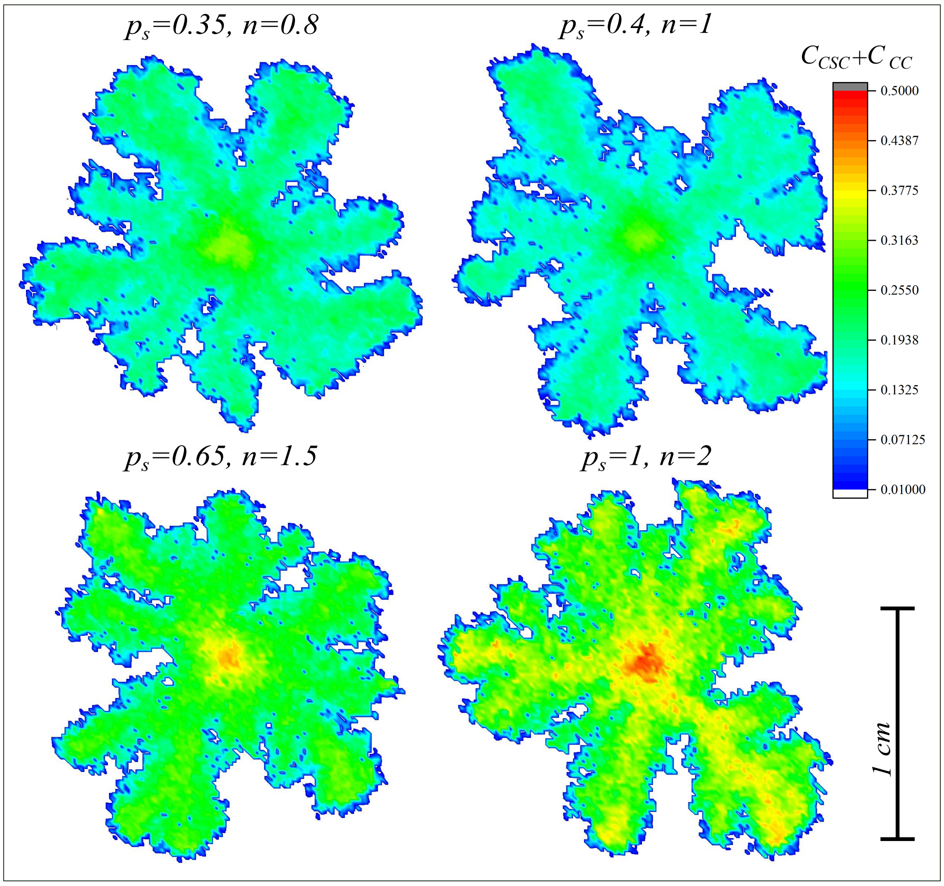

FIG. 3: The same behavior of tumors can be the result of different conditions. The probability p_s increases invasion, while the nutrient density has adverse effect on the invasion. Thus, if we increase/decrease both, their effect may compensate each other and the tumor behavior can remain unchanged.

how close a shape is to a circle, and is defined by

$$Circularity(r) = \frac{\text{area which is filled by shape between } r \text{ and } r + dr}{\text{area of ring wtih radius equal to } r \text{ with thickness of } dr}$$

Based on the previous studies, tumors with larger values of the probability p_s should have more regular shapes than those with lower values [8]. Our results indicate, however, a completely different behavior. In addition to the fractal analysis, circularity indicates that irregularities increase for each tumor during its growth and for tumors with the same area, those with larger p_s have more irregular shapes. Circularity in tumors of different sizes, as well as for different tumors but with the same mass, are shown in Fig. 13

We also present the cells' distribution in various tumors under various conditions.

To test the various assumptions of the model and their efect on the main results, we carried out extensive simulations with various scenarios. In what follows we describe the results.

(a) Clearly, alternative boundary conditions and oxygen supply systems can be considered, and the model is fully capable of adapting them. Thus, we carried out simulations in which we varied the density of oxygen at the perimeter of the circle with a radius of 1 cm. Figure 8 shows that the main results for the relation between D_f and p_s are preserved.

(b) Regarding the radius of the circles (see the text of the paper), we varied the size of the medium in which the tumor grows. The results are shown in Fig. 9, indicating that they do not depend on radius of the circle.

(c) As for the structure of the oxygen supply system, We carried out simulations in which the same disc (circle) was considered, but instead of supplying the oxygen from the outside of the disc, we used a lattice of vessels that were separated by 0.2 mm and supplied the oxygen to the tumor. Then, the density of oxygen at such units was updated to 1 with a fixed certain rate. Equal numbers of such units with a random spatial distribution in the disc were used. We also simulated the case in which each unit directly acquires its oxygen. In all the cases, other rules for

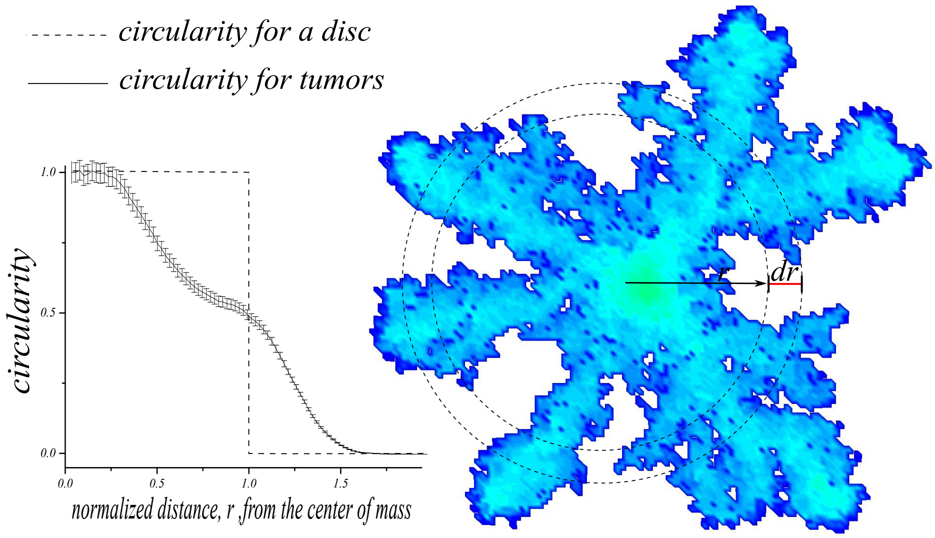

FIG. 4: Definition of circularity as the area covered by a tumor divided by the area of the corresponding ring (right). Circularity for a disc is equal to 1 for distances less than its radius and vanishes for larger distances (dashed line on the left). For tumors, the distance from the center of mass is normalized by the radius of a disc with the same area. Shown is the result averaged over 100 tumors.

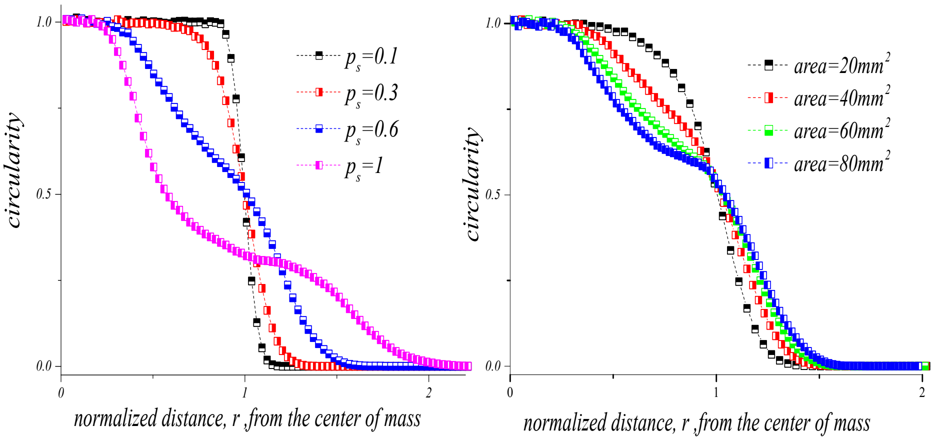

FIG. 5: Right panel: Tumors shape during growth can also become irregular. This result conforms with increasing the invasive behavior of tumor with time [9]. Left panel: Circularity of various tumors that indicates that tumors with larger probability p_s have more irregular shapes. The error bars are smaller than the symbol size.

nutrient evolution, such as oxygen diffusion, remained unchanged. But, as Fig. 10 shows, the main results remained unchanged.

(d) In the main simulations we used the reported value of oxygen diffusion cofficient β [3, 10], but the results will not change by lower values of β. Figure 11 shows that our results do not depend on β.

(e) The mobility of different kinds of cells is not the same, of course. We assumed the diffusion coefficient of the cells to be the same, but as Fig. 12 indicates, the main results do not depend on the differences between the diffusion coefficients of various cells.

(f) Regarding the oxygen consumption rate, α: the CSCs and CCs are assumed to have the same rate of oxygen consumption, but when we changed the rates for every kind of cell, the results remained unchanged, as Fig. 13 demonstrates it.

(g) The CSCs and CCs are assumed to have the same internal energy threshold u_p for duplication, and equal rates of crossing the S, G_2 and M phases in the cell cycle, R_m. But, changing the proliferation activity of the cells does

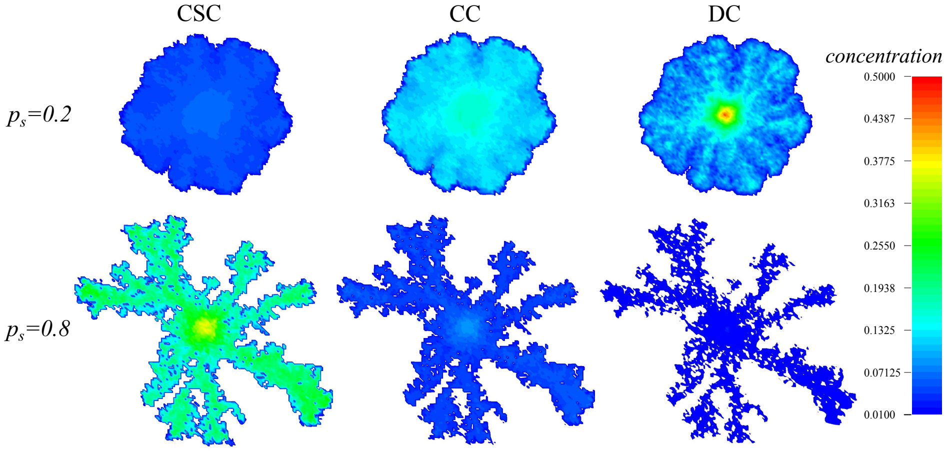

FIG. 6: The cell distribution under the same external condition for various tumors.

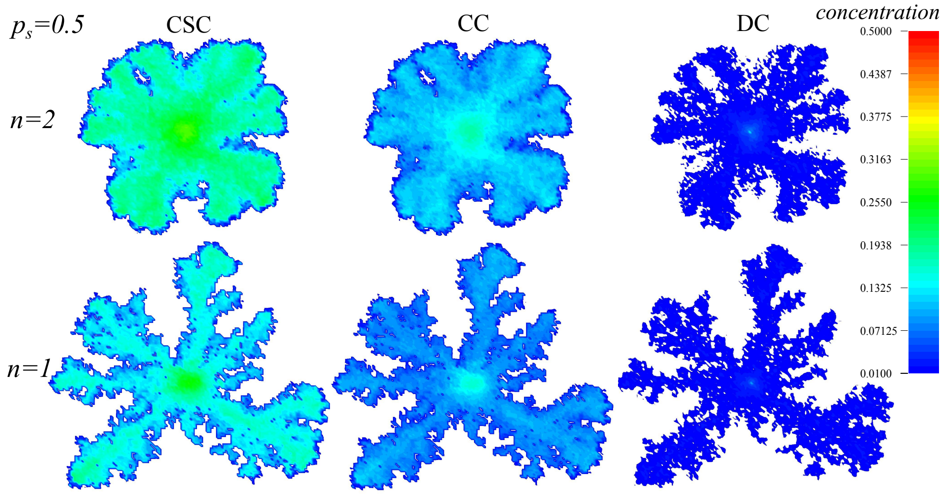

FIG. 7: The cell distribution for the same tumor under various external condition.

not change the main result; see Fig. 14.

(h) We assume that the dead cells remain inactive in the medium. But, even if we eliminate them after their death, the main results would be unchanged. This is shown in Fig. 15.

I. PARAMETERS

To give a comprehend view to readers, here we present all parameters we used in our model.

[1] A. Brú, J. M. Pastor, I. Fernaud, I. Brú, S. Melle, and C. Berenguer, Physical Review Letters **81**, 4008 (1998).

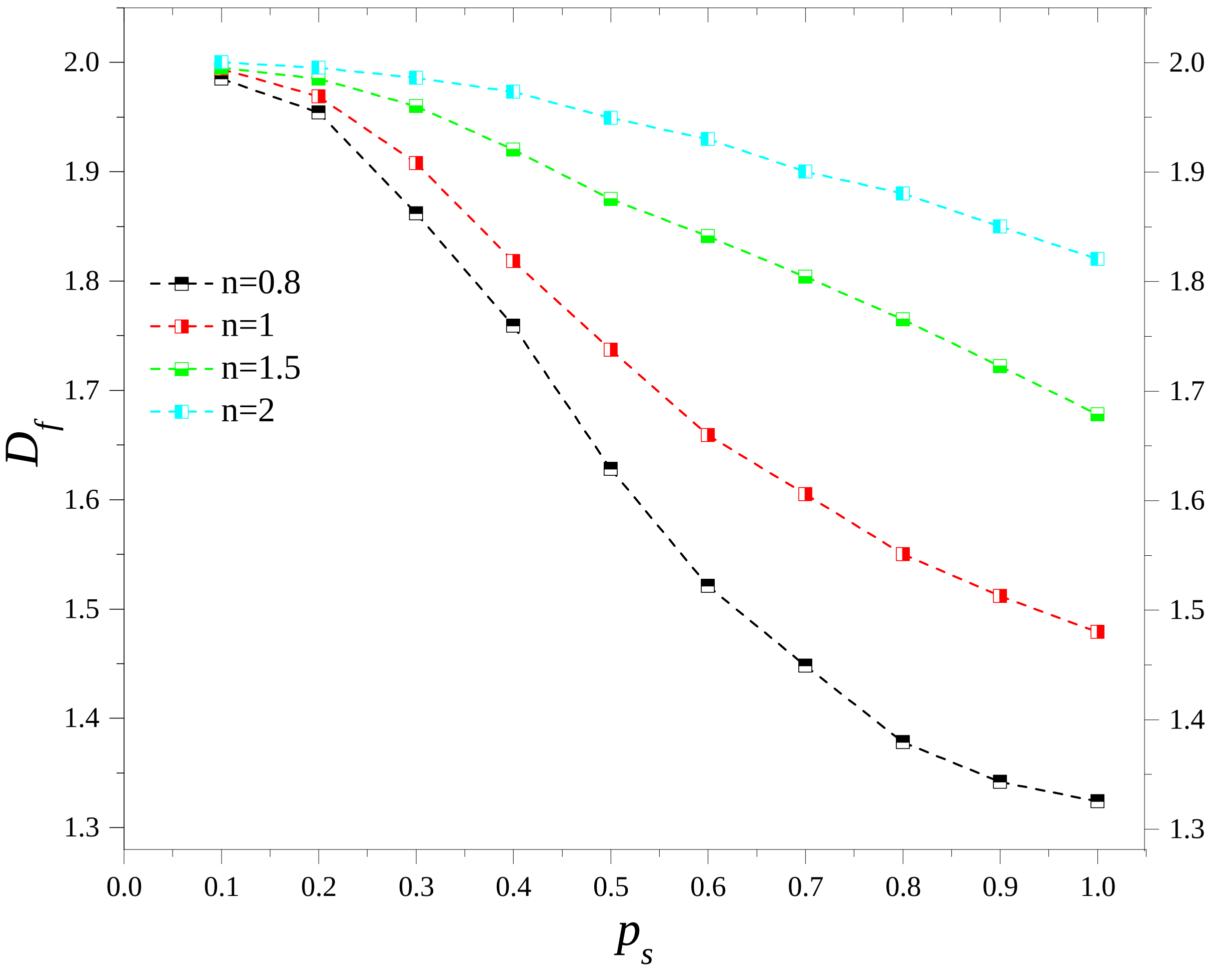

FIG. 8: Effect of oxygen density on the tumor behavior. The relation between malignancy of the tumor and the number of the CSCs does not depend on the oxygen density at the perimeter of medium. As long as the cells could find enough oxygen to duplicate and create tumors, the tumor's behavior depends on the number of the CSCs.

[2] A. Brú, S. Albertos, J. L. Subiza, J. L. García-Asenjo, and I. Brú, Biophysical journal **85**, 2948 (2003).
[3] A. R. Anderson, Mathematical Medicine and Biology **22**, 163 (2005).
[4] A. R. Anderson, A. M. Weaver, P. T. Cummings, and V. Quaranta, Cell **127**, 905 (2006).
[5] P. Gerlee and A. R. Anderson, Physical Review E **75**, 051911 (2007).
[6] P. Gerlee and A. R. Anderson, Mathematical biosciences and engineering: MBE **7**, 385 (2010).
[7] A. R. Anderson and V. Quaranta, Nature Reviews Cancer **8**, 227 (2008).
[8] H. Enderling, L. Hlatky, and P. Hahnfeldt, Breast **11**, 200 (2013).
[9] D. Hanahan and R. A. Weinberg, cell **100**, 57 (2000).
[10] A. Sottoriva, J. J. Verhoeff, T. Borovski, S. K. McWeeney, L. Naumov, J. P. Medema, P. M. Sloot, and L. Vermeulen, Cancer research **70**, 46 (2010).
[11] N. K. Haass, K. A. Beaumont, D. S. Hill, A. Anfosso, P. Mrass, M. A. Munoz, I. Kinjyo, and W. Weninger, Pigment cell & melanoma research **27**, 764 (2014).
[12] D. Bray, *Cell movements: from molecules to motility* (Garland Science, 2001).
[13] J. MacDougall and M. McCabe, Nature **215**, 1173 (1967).
[14] J. J. Casciari, S. V. Sotirchos, and R. M. Sutherland, Journal of cellular physiology **151**, 386 (1992).

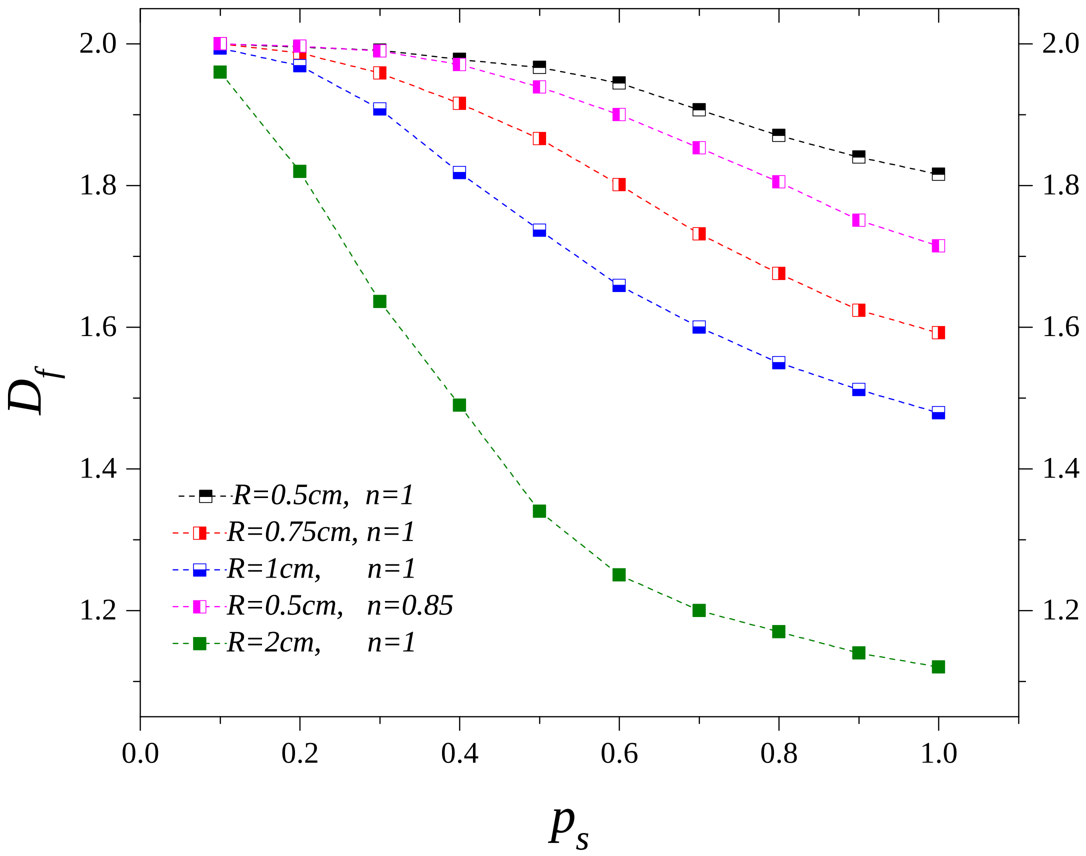

FIG. 9: Tumor's behavior in mediums with various radii and oxygen density. When the medium's radius decreases, the nutrient source becomes more convenient and the tumors would be less aggressive, but under the same conditions, the number of the CSCs increases the invasive behavior. Moreover, when oxygen density at the border of medium decreases, the invasive behavior becomes stronger while a larger number of the CSCs leads to stronger invasive behavior.

TABLE I: Parameters used in the model and their definition and value.

Parameter	definition	Value
χ	energy obtain rate	1 (estimated)
γ	energy consumption rate	0.1 (estimated)
b_m	mitosis energy threshold	8 (estimated)
R_m	mitosis rate	0.05 [11]
R_a	apoptosis rate	0.05 (estimated)
D	Cells random motion constant	10^{-10}cm^2/s [3, 12]
β	oxygen diffusion constant in tissue	10^{-5} cm^2/s [13]
α	oxygen consumption rate	6.65×10^{-17} mol cell^{-1}s^{-1} [3, 14]

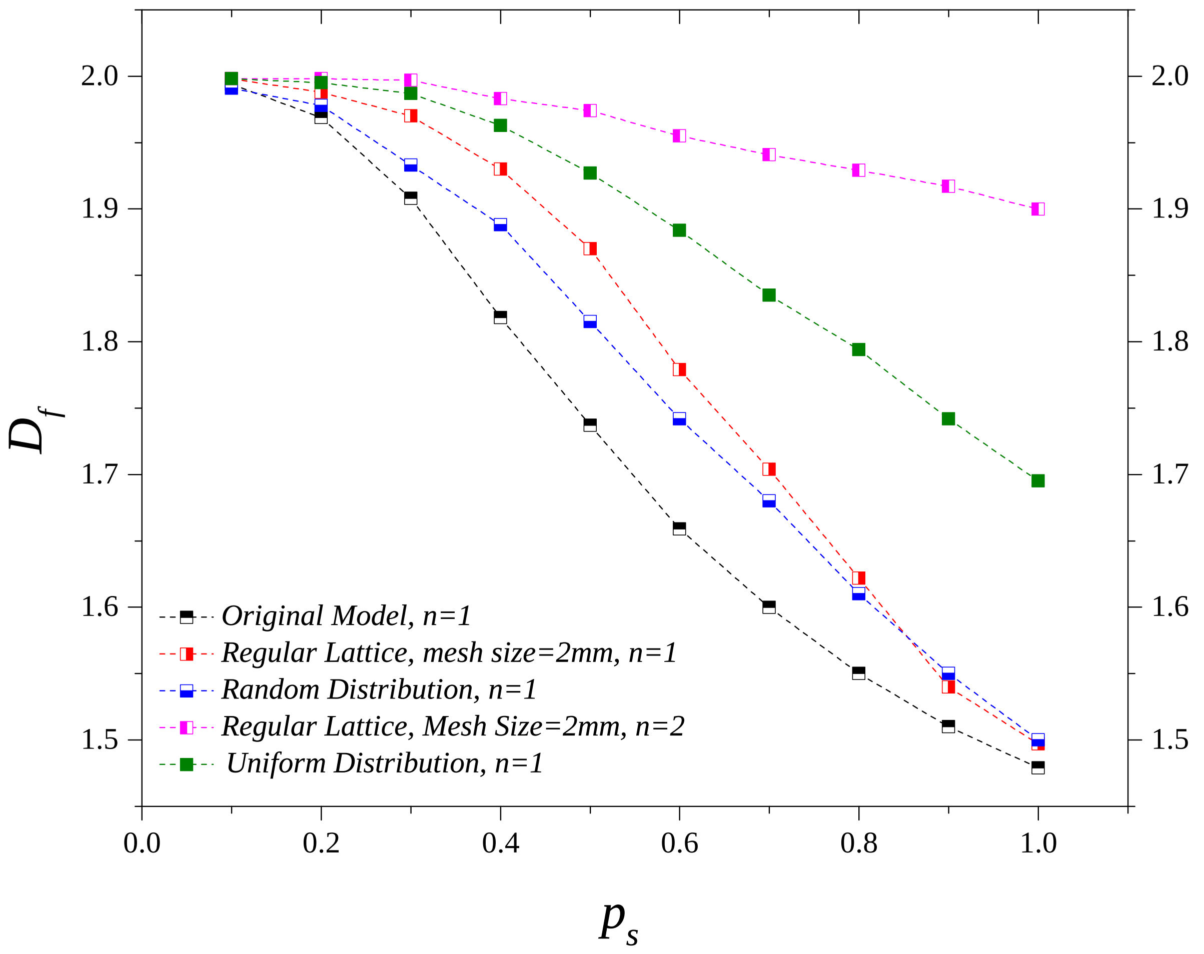

FIG. 10: The behavior of system under various structures of the nutrient supply. Instead of the perimeter of the medium, some units inside it act as the source of oxygen. The density of oxygen in such units is updated to a fixed value (in this case, 1 and 2), similar to the density of oxygen at the perimeter of medium in the base model. Similar to the original model, oxygen diffuses into other units that could be the nodes of a uniform lattice with a lattice unit of 2 mm. Moreover, we considered the same number of the units with a random spatial distribution in the medium. Finally, for each unit we considered a direct oxygen supply. As the figure indicates, the main results do not depend on the structure of the oxygen supply system.

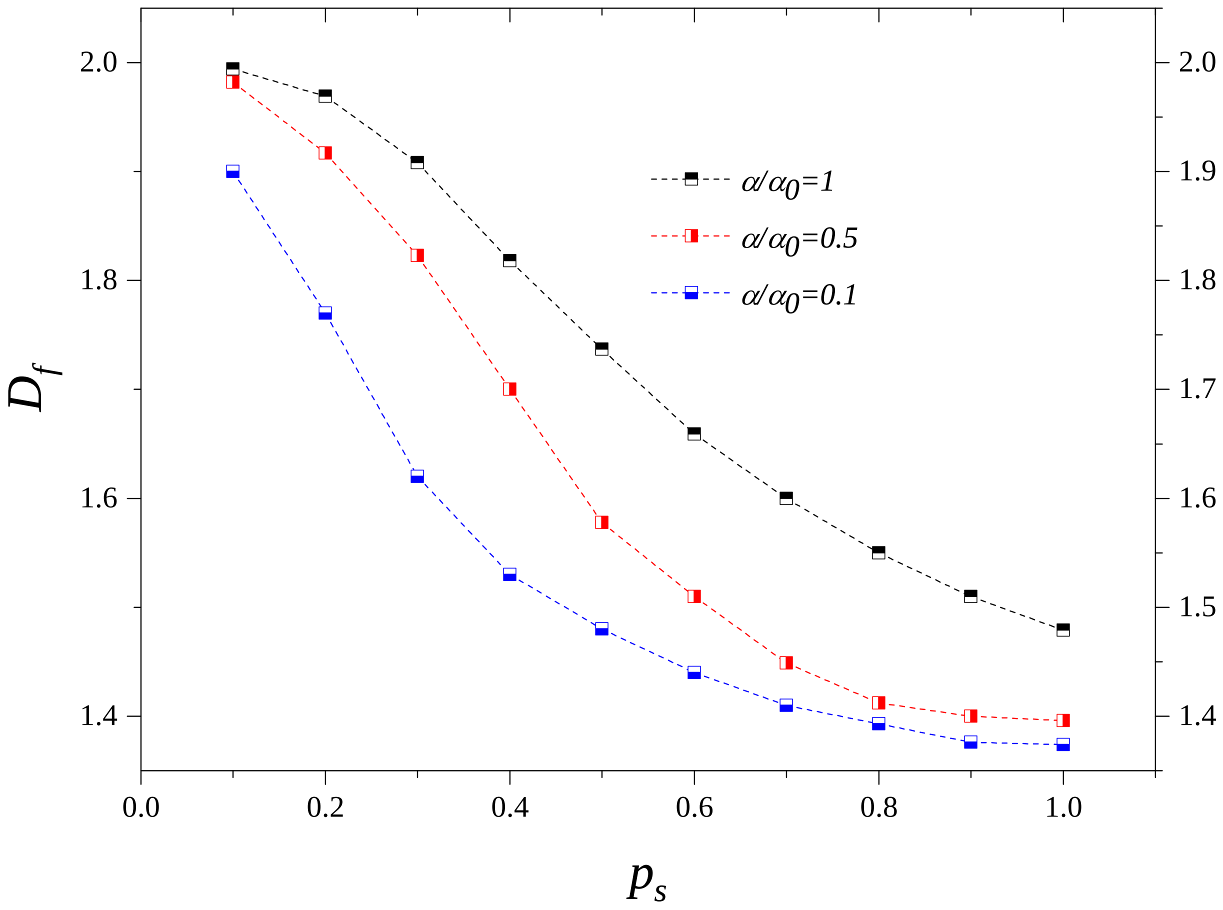

FIG. 11: The behavior of the tumors in a medium with various oxygen diffusion coefficients. The main results presented in the text of the paper do not change even in a mediums with low permeability of oxygen.

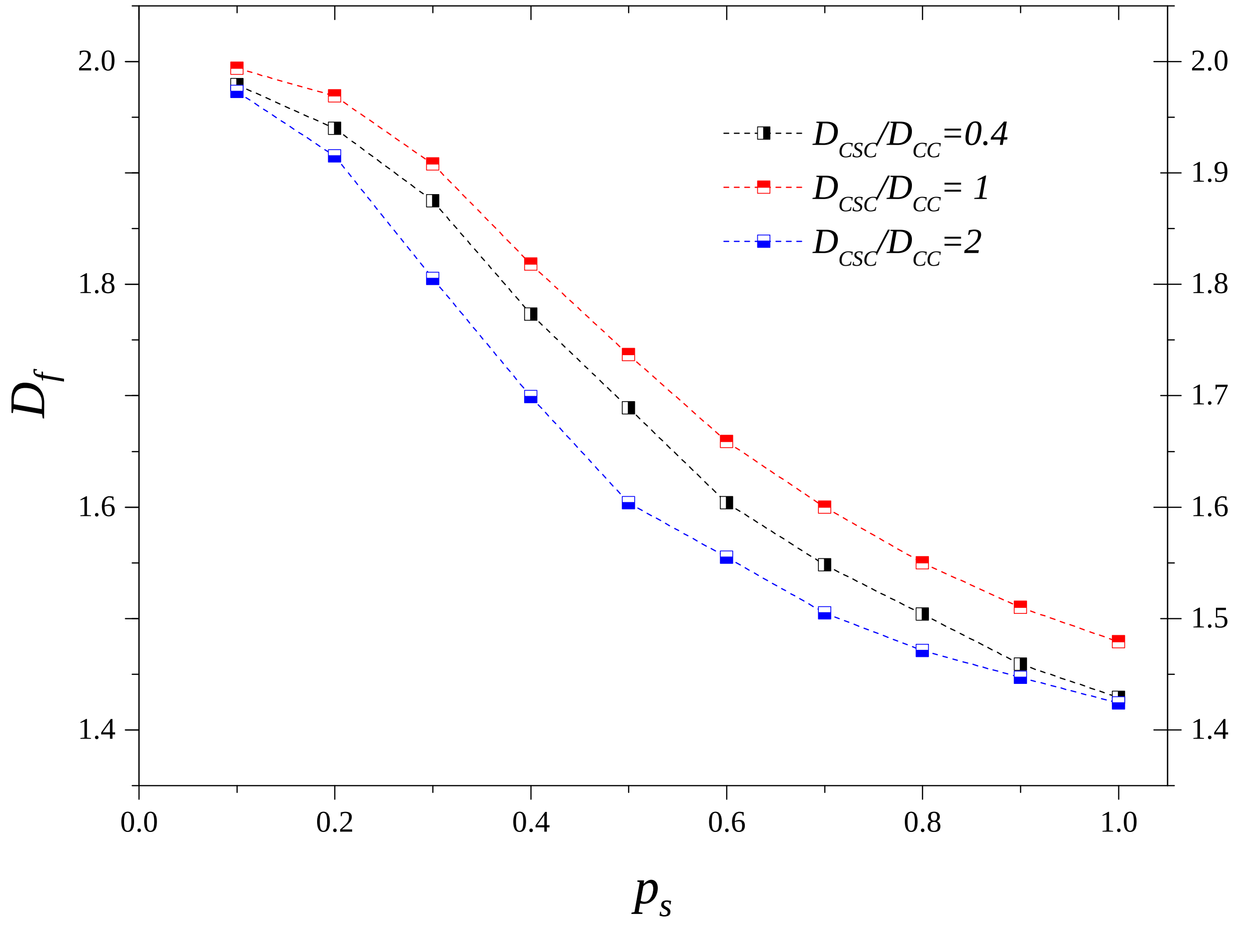

FIG. 12: The behavior of the tumors with various scenarios for the cells' motility. Although the CSCs usually have larger motility, our results do not depend on the motility of the CSCs and CCs.

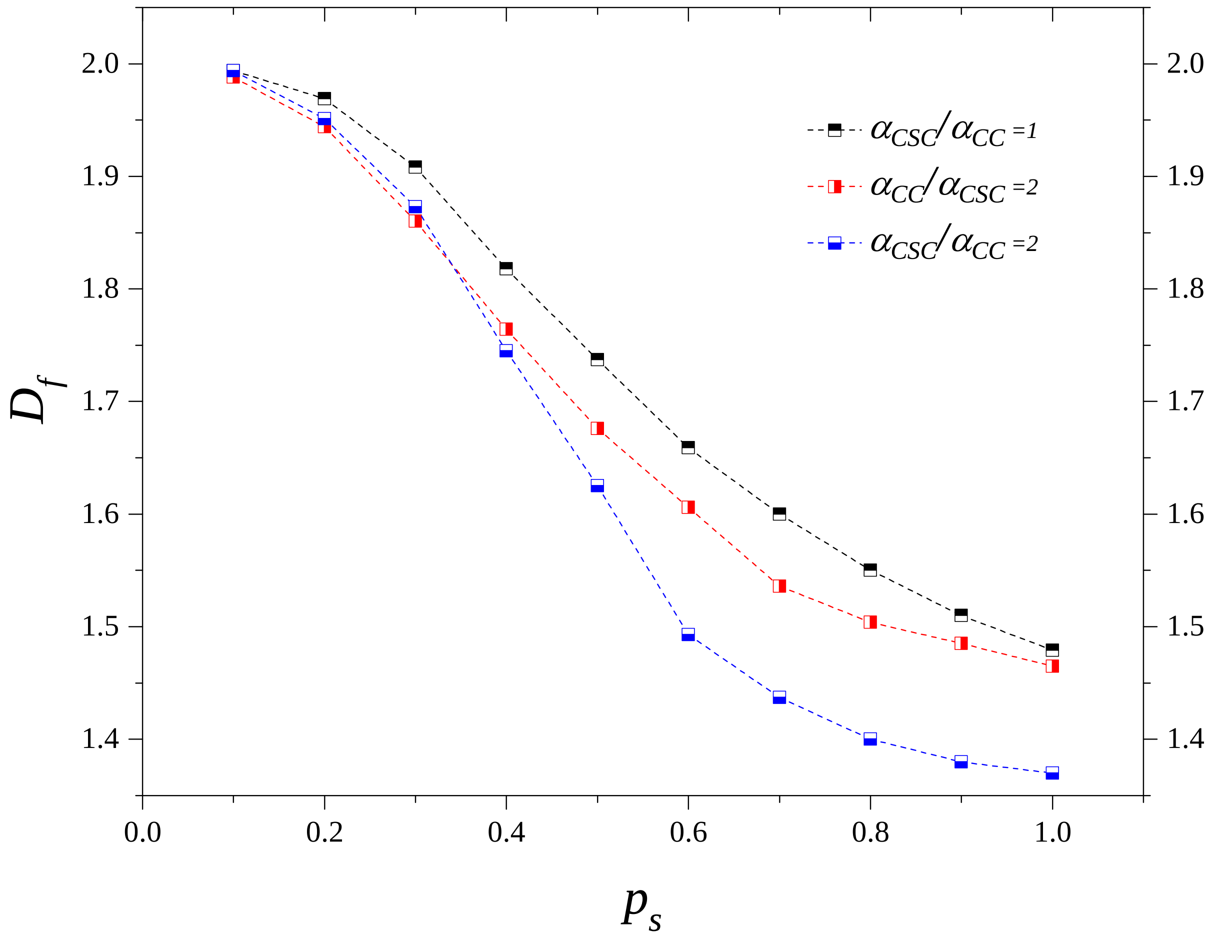

FIG. 13: Effect of oxygen consumption rate of cells on the tumor behavior. If one kind of the cells has larger consumption rate, then the fractal dimension of the tumor would be different, but the relation between malignancy and the number of the CSCs remains unchanged.

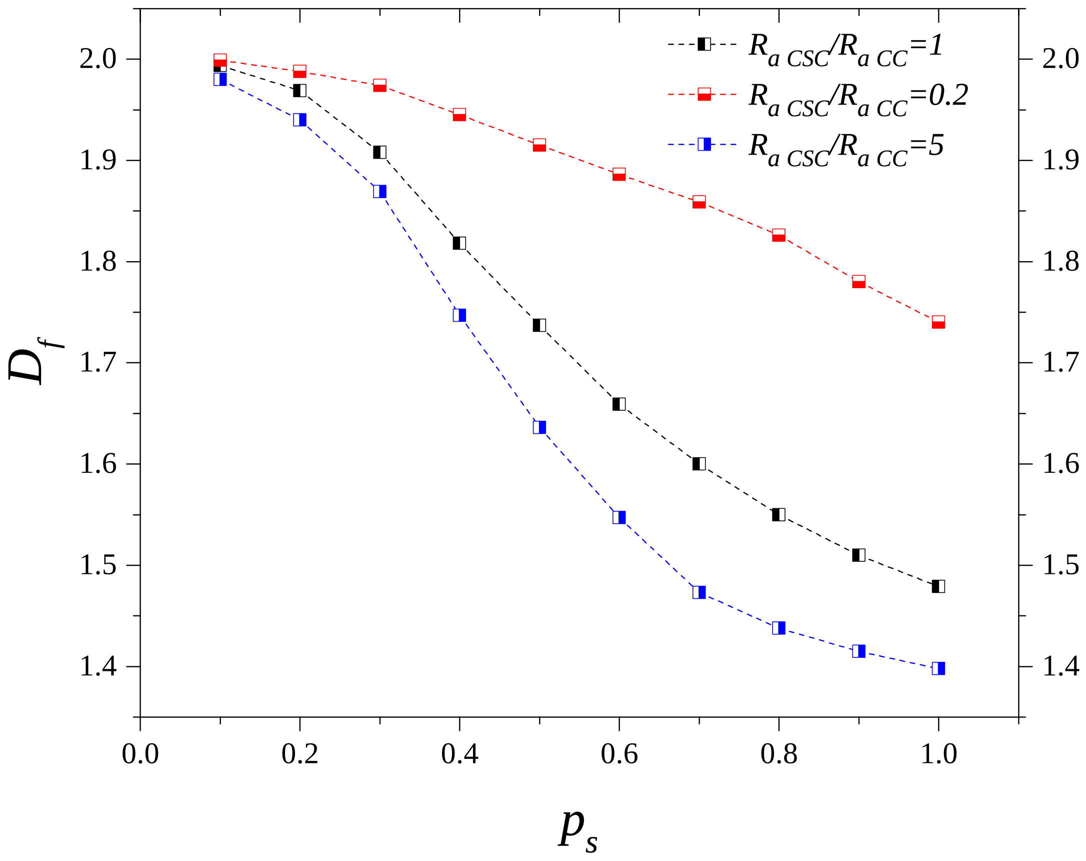

FIG. 14: Effect of the cells' duplication rates on the behavior of the tumor. Whether the CSCs duplicate faster (five times) or slower (five times) than the CCs does not change the main results.

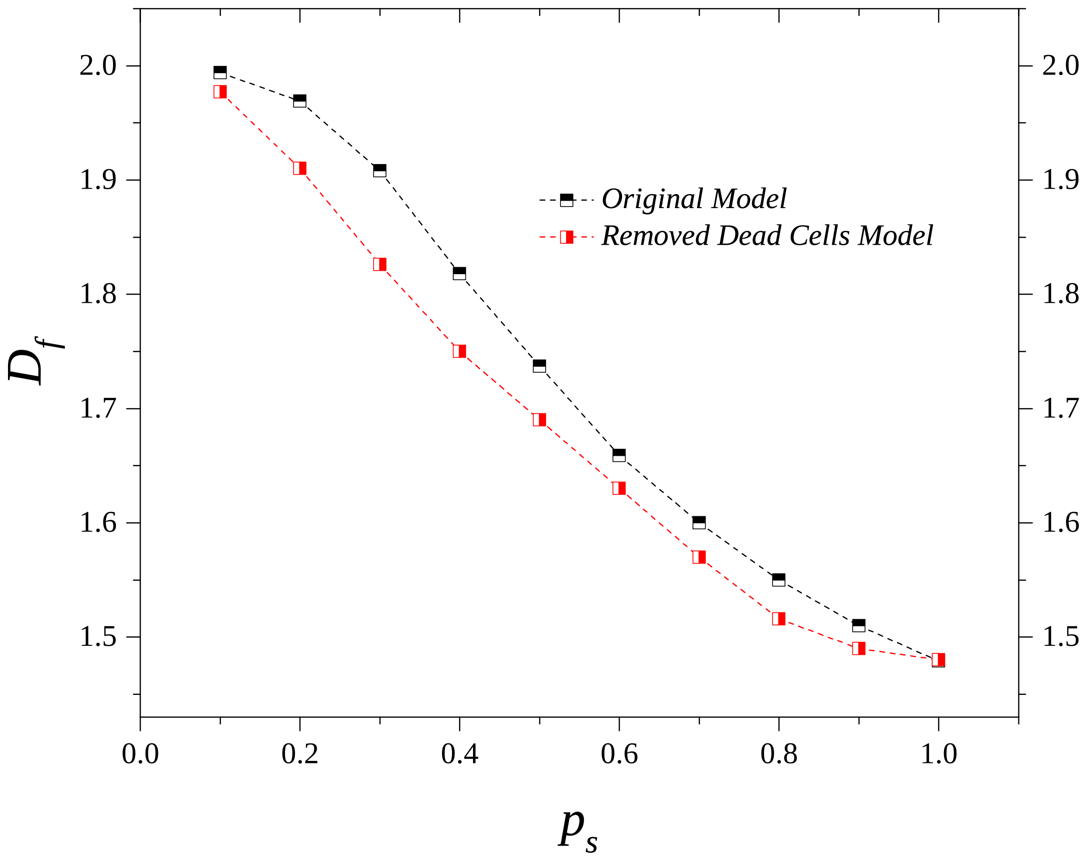

FIG. 15: Effect of the dead cells' destiny on the tumor behavior. We considered two possible scenarios for the fate of the dead cells. But, because of their marginal role, their existence in the model (or removal) has no considerable role in the tumor's behavior.
